# 5,5,7,12,14,14-Hexamethyl-1,8-diaza-4,11-diazo­niacyclo­tetra­deca-4,11-diene dichloride trihydrate

**DOI:** 10.1107/S1600536812016649

**Published:** 2012-04-21

**Authors:** Wafiuddin Ismail, Bohari M. Yamin, Jean-Claude Daran

**Affiliations:** a Low Carbon Energy Research Group, School of Chemical Sciences and Food Technology, Universiti Kebangsaan Malaysia, UKM 43500 Bangi Selangor, Malaysia; b Laboratoire de Chimie de Coordination, UPR5241, 205, route de Narbonne 31077, Toulose Cedex 04, France

## Abstract

In the title compound, C_16_H_34_N_4_^2+^·2Cl^−^·3H_2_O, the two protonated N atoms in the macrocyclic ring of the dication are located at diagonally opposite positions. There are two intramolecular N—H⋯N hydrogen bonds in the cation. The crystal structure features O—H⋯Cl, O—H⋯O, C—H⋯Cl and N—H⋯Cl hydrogen bonds.

## Related literature

For related structures, see: Bi *et al.* (2008 ▶); He *et al.* (2010 ▶); Heeg *et al.* (1981 ▶); Heinlein & Tebbe (1985 ▶); Kennedy *et al.* (2011 ▶); Rohovec *et al.* (1999 ▶). For bond-length data, see: Allen *et al.* (1987 ▶). For the preparation, see: Curtis & Hay (1966 ▶); Curtis *et al.* (1975 ▶). For applications of macrocyclic compounds, see: Mittal *et al.* (2008 ▶); Yatsimirskii (1990 ▶).
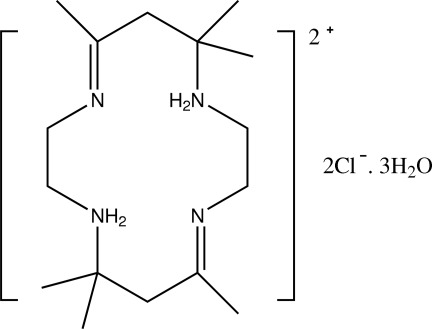


## Experimental

### 

#### Crystal data

C_16_H_34_N_4_^2+^·2Cl^−^·3H_2_O
*M* *_r_* = 407.42
Triclinic, 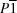

*a* = 8.576 (4) Å
*b* = 10.735 (4) Å
*c* = 13.438 (6) Å
α = 73.752 (9)°
β = 86.085 (9)°
γ = 71.886 (8)°
*V* = 1128.7 (8) Å^3^
*Z* = 2
Mo *K*α radiation
μ = 0.31 mm^−1^
*T* = 298 K
0.36 × 0.14 × 0.13 mm

#### Data collection

Bruker SMART APEX CCD area-detector diffractometer
Absorption correction: multi-scan (*SADABS*; Bruker, 2000 ▶) *T*_min_ = 0.897, *T*_max_ = 0.961
11977 measured reflections
3987 independent reflections
2878 reflections with *I* > 2σ(*I*)
*R*_int_ = 0.051

#### Refinement

*R*[*F* ^2^ > 2σ(*F* ^2^)] = 0.055
*wR*(*F* ^2^) = 0.136
*S* = 1.07
3987 reflections
248 parameters
H atoms treated by a mixture of independent and constrained refinement
Δρ_max_ = 0.28 e Å^−3^
Δρ_min_ = −0.16 e Å^−3^

### 

Data collection: *SMART* (Bruker, 2000 ▶); cell refinement: *SAINT* (Bruker, 2000 ▶); data reduction: *SAINT*; program(s) used to solve structure: *SHELXTL* (Sheldrick, 2008 ▶); program(s) used to refine structure: *SHELXTL*; molecular graphics: *SHELXTL*; software used to prepare material for publication: *SHELXTL*, *PARST* (Nardelli, 1995 ▶) and *PLATON* (Spek, 2009 ▶).

## Supplementary Material

Crystal structure: contains datablock(s) global, I. DOI: 10.1107/S1600536812016649/hg5196sup1.cif

Structure factors: contains datablock(s) I. DOI: 10.1107/S1600536812016649/hg5196Isup2.hkl

Additional supplementary materials:  crystallographic information; 3D view; checkCIF report

## Figures and Tables

**Table 1:** Hydrogen-bond geometry (Å, °)

*D*—H⋯*A*	*D*—H	H⋯*A*	*D*⋯*A*	*D*—H⋯*A*
N2—H2*N*2⋯N1	0.90 (3)	2.02 (3)	2.732 (3)	135.6 (18)
N4—H2*N*4⋯N3	0.86 (3)	2.03 (3)	2.740 (3)	140 (2)
O1*W*—H2*W*1⋯Cl1	0.82	2.54	3.343 (4)	167
O2*W*—H2*W*2⋯Cl1	0.82	2.51	3.324 (3)	168
O3*W*—H1*W*3⋯Cl1	0.83	2.53	3.298 (3)	156
O3*W*—H2*W*3⋯Cl2	0.82	2.32	3.138 (3)	175
N2—H1*N*2⋯Cl1	0.92 (3)	2.28 (3)	3.201 (3)	177 (3)
N4—H1*N*4⋯Cl2	0.93 (3)	2.27 (3)	3.178 (3)	167 (2)
O1*W*—H1*W*1⋯Cl2^i^	0.82	2.49	3.282 (3)	163
O2*W*—H1*W*2⋯O3*W*^i^	0.82	2.16	2.955 (4)	161
C9—H9*B*⋯Cl2^i^	0.97	2.75	3.709 (3)	172

Symmetry code: (i) 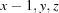.

**Fig. 1.**
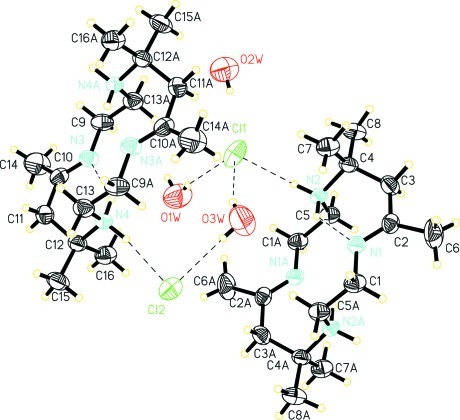
The molecular structure of (I), with displacement ellipsods are drawn at the 50% probability level. The dashed line indicate intramolecular hydrogen bond. The unlabelled atoms are symmetrically generated (1-x, 1-y,-z).

## supplementary crystallographic information

### Comment

The structures of tetraazacyclotetradeca-4,11-diene macrocyclic complexes with metal such as cobalt, nickel, zinc have been extensively studied (Heeg *et al.*, 1981; He *et al.*, 2010; Heinlein *et al.*, 1985). Some macrocyclic compounds and their complexes have been applied as ionophores for metal ions determination and catalyst for several reactions (Mittal *et al.*, 2008; Yatsimirskii 1990). However, the structure of the macrocyclic salts are still less reported. So far, the macrocyclic salts with perchlorate, bromide and iodide anion have been reported (Rohovec *et al.*, 1999; Kennedy *et al.*, 2011; Bi *et al.*, 2008). The unit-cell parameters for the bromide and iodide salt are similar and the two salts are indeed isostructural. The title compound (I) is similar to those salts but the presence of trihydrate water molecules caused the unit-cell parameters to be different. The unit cell of the salt consists of symmetrically generated macrocyclic dication, two chloride anions atoms and three water molecules of crystallization (Fig.1). The bond lengths are in normal ranges (Allen *et al.* 2003) and comparable to those in the bromide and iodide salts. There are seven intramolecular hydrogen bonds, two of them are in the macrocyclic ring N2-H1N2..N1 and N4-H2N4..N3 and the other five, O1-H2W1..Cl1, O2-H2W2..Cl1, O3-H1W3..Cl1, O3W-H2W3..Cl2 and N4-H1N4..Cl2 are formed between chlorine atom and hydrogen atom of the water molecule and nitrogen atom of the macrocyclic ring. In the crystal structure, the molecules are linked by intermolecular hydrogen bond, O1W-H1W1..Cl2, O2W-H1W2..O3W and C9—H9B..Cl2 (symmetry as in table 2) together with the OW—H..Cl and N—H..Cl intramolecular hydrogen bonds.

### Experimental

All solvents and chemicals were of analytical grade and were used without purification. The macrocylic compound was prepared according to the literature methods (Curtis *et al.*, 1966; Curtis *et al.*, 1975) but with the addition of stoichiometric amounts of ammonium chloride (0.01 mol, 0.534 g) and ethylenediamine (0.01 mol, 0.601 g) in 30 ml acetone. Single crystals were obtained from the solution after one day of evaporation (yield 82%, m.p 372.1–372.8 K). IR(KBr)v_max_, cm^-1^: 1667.1 (C=N); 3468.2 (NH); 1227.9 (C—N); 3012.4(CH_3_). Elemental analysis: Calc. for C_16_H_40_N_4_O_3_ Cl_2_(C: 47.2; N: 13.7; H: 9.4%) Found (C: 46.9; N: 13.26; H: 9.3%). ^1^H NMR (p.p.m.,CD_3_OH), δ_H_: 1.5 (s,12*H*,*C*-(CH_3_)_2_); 2.1 (s,6*H*,*C*—CH_3_); 2.8 (s,4*H*,*C*—CH_2_—C); 3.3 (m,4*H*,CH_2_—CH_2_); 3.8 (m,4*H*,CH_2_—CH_2_); 5.1 (s,2*H*,NH_2_). ^13^C NMR (p.p.m.,CD_3_OH), δ_C_: 175.9 (N=C—C); 58.4 (C—C—N); 47.4 (C=N—CH_2_); 43.91 (N—CH_2_—C); 40.0 (C—CH_2_—C); 23.47 (N=C—CH_3_); 20.65 (C-(CH_3_)_2_).

### Refinement

All H atoms attached to C atoms were fixed geometrically and treated as riding with C—H= 0.96 Å for methyl or 0.97 Å for methylene groups with *U*_iso_(H)=1.2*U*_eq_(C) and 1.5U_eq_(C) for methylene and methyl groups respectively. The hydrogen atoms attached to nitrogen and oxygen atoms were located from the Fourier difference map and refined isotropiclly. The rotating model was applied in the refinement of the methyl hydrogen atoms.

### Crystal data

**Table:**

C_16_H_34_N_4_^2+^·2Cl^−^·3H_2_O	*Z* = 2
*M**_r_* = 407.42	*F*(000) = 444
Triclinic, *P*1	*D*_x_ = 1.199 Mg m^−^^3^
Hall symbol: -P 1	Melting point = 372.1–372.8 K
*a* = 8.576 (4) Å	Mo *K*α radiation, λ = 0.71073 Å
*b* = 10.735 (4) Å	Cell parameters from 2072 reflections
*c* = 13.438 (6) Å	θ = 1.6–25.0°
α = 73.752 (9)°	µ = 0.31 mm^−^^1^
β = 86.085 (9)°	*T* = 298 K
γ = 71.886 (8)°	Block, colourless
*V* = 1128.7 (8) Å^3^	0.36 × 0.14 × 0.13 mm

### Data collection

**Table:**

Bruker SMART APEX CCD area-detector diffractometer	3987 independent reflections
Radiation source: fine-focus sealed tube	2878 reflections with *I* > 2σ(*I*)
Graphite monochromator	*R*_int_ = 0.051
Detector resolution: 83.66 pixels mm^-1^	θ_max_ = 25.0°, θ_min_ = 1.6°
ω scan	*h* = −10→10
Absorption correction: multi-scan (*SADABS*; Bruker, 2000)	*k* = −12→12
*T*_min_ = 0.897, *T*_max_ = 0.961	*l* = −15→15
11977 measured reflections	

### Refinement

**Table:**

Refinement on *F*^2^	Primary atom site location: structure-invariant direct methods
Least-squares matrix: full	Secondary atom site location: difference Fourier map
*R*[*F*^2^ > 2σ(*F*^2^)] = 0.055	Hydrogen site location: inferred from neighbouring sites
*wR*(*F*^2^) = 0.136	H atoms treated by a mixture of independent and constrained refinement
*S* = 1.07	*w* = 1/[σ^2^(*F*_o_^2^) + (0.0559*P*)^2^ + 0.1922*P*] where *P* = (*F*_o_^2^ + 2*F*_c_^2^)/3
3987 reflections	(Δ/σ)_max_ < 0.001
248 parameters	Δρ_max_ = 0.28 e Å^−^^3^
0 restraints	Δρ_min_ = −0.16 e Å^−^^3^

### Special details

**Table:**

Geometry. All e.s.d.'s (except the e.s.d. in the dihedral angle between two l.s. planes) are estimated using the full covariance matrix. The cell e.s.d.'s are taken into account individually in the estimation of e.s.d.'s in distances, angles and torsion angles; correlations between e.s.d.'s in cell parameters are only used when they are defined by crystal symmetry. An approximate (isotropic) treatment of cell e.s.d.'s is used for estimating e.s.d.'s involving l.s. planes.
Refinement. Refinement of *F*^2^ against ALL reflections. The weighted *R*-factor *wR* and goodness of fit *S* are based on *F*^2^, conventional *R*-factors *R* are based on *F*, with *F* set to zero for negative *F*^2^. The threshold expression of *F*^2^ > σ(*F*^2^) is used only for calculating *R*-factors(gt) *etc*. and is not relevant to the choice of reflections for refinement. *R*-factors based on *F*^2^ are statistically about twice as large as those based on *F*, and *R*- factors based on ALL data will be even larger.

### Fractional atomic coordinates and isotropic or equivalent isotropic displacement parameters (Å^2^)

**Table:**

	*x*	*y*	*z*	*U*_iso_*/*U*_eq_	
Cl1	0.54889 (10)	0.63892 (9)	0.26242 (6)	0.0731 (3)	
Cl2	0.91229 (9)	0.98055 (8)	0.24245 (6)	0.0552 (2)	
O1W	0.2897 (3)	0.9092 (3)	0.3252 (2)	0.1010 (9)	
H1W1	0.2055	0.9197	0.2942	0.151*	
H2W1	0.3523	0.8513	0.2998	0.151*	
O2W	0.2391 (3)	0.5665 (3)	0.18015 (19)	0.0887 (8)	
H1W2	0.1576	0.6126	0.2037	0.133*	
H2W2	0.3136	0.5769	0.2095	0.133*	
O3W	0.9133 (3)	0.6822 (2)	0.26157 (19)	0.0869 (8)	
H1W3	0.8192	0.6909	0.2442	0.130*	
H2W3	0.9170	0.7602	0.2532	0.130*	
N1	1.1625 (2)	0.3762 (2)	0.45434 (15)	0.0365 (5)	
N2	0.8331 (3)	0.4327 (2)	0.42420 (16)	0.0336 (5)	
H1N2	0.754 (3)	0.494 (3)	0.377 (2)	0.049 (8)*	
H2N2	0.920 (3)	0.463 (2)	0.4218 (17)	0.032 (7)*	
N3	0.3084 (2)	1.0996 (2)	0.03415 (16)	0.0394 (5)	
N4	0.6102 (3)	1.1063 (2)	0.08282 (16)	0.0335 (5)	
H1N4	0.688 (3)	1.061 (3)	0.136 (2)	0.054 (8)*	
H2N4	0.542 (3)	1.063 (2)	0.0808 (17)	0.026 (6)*	
C1	1.2999 (3)	0.4269 (3)	0.4597 (2)	0.0409 (6)	
H1A	1.3631	0.4267	0.3971	0.049*	
H1B	1.3712	0.3675	0.5182	0.049*	
C2	1.1821 (3)	0.2496 (3)	0.47770 (18)	0.0365 (6)	
C3	1.0377 (3)	0.2016 (3)	0.4681 (2)	0.0421 (6)	
H3A	0.9935	0.1775	0.5367	0.051*	
H3B	1.0785	0.1188	0.4460	0.051*	
C4	0.8963 (3)	0.2984 (2)	0.39511 (19)	0.0365 (6)	
C5	0.7616 (3)	0.4304 (3)	0.52835 (19)	0.0392 (6)	
H5A	0.8443	0.3738	0.5815	0.047*	
H5B	0.6717	0.3918	0.5368	0.047*	
C6	1.3381 (3)	0.1384 (3)	0.5155 (3)	0.0673 (9)	
H6A	1.4014	0.1686	0.5548	0.101*	
H6B	1.4000	0.1158	0.4573	0.101*	
H6C	1.3131	0.0595	0.5587	0.101*	
C7	0.9524 (3)	0.3337 (3)	0.2835 (2)	0.0477 (7)	
H7A	1.0376	0.3754	0.2794	0.072*	
H7B	0.8613	0.3959	0.2397	0.072*	
H7C	0.9936	0.2522	0.2612	0.072*	
C8	0.7565 (3)	0.2372 (3)	0.4025 (2)	0.0503 (7)	
H8A	0.7280	0.2089	0.4738	0.076*	
H8B	0.7904	0.1600	0.3746	0.076*	
H8C	0.6629	0.3043	0.3637	0.076*	
C9	0.2026 (3)	1.0191 (3)	0.0267 (2)	0.0434 (7)	
H9A	0.1299	1.0660	−0.0334	0.052*	
H9B	0.1355	1.0093	0.0878	0.052*	
C10	0.2476 (3)	1.2250 (3)	0.02649 (19)	0.0392 (6)	
C11	0.3598 (3)	1.3067 (3)	0.0326 (2)	0.0446 (7)	
H11A	0.2945	1.3883	0.0511	0.054*	
H11B	0.4019	1.3353	−0.0360	0.054*	
C12	0.5058 (3)	1.2355 (2)	0.10895 (19)	0.0385 (6)	
C13	0.6969 (3)	1.1195 (3)	−0.01763 (19)	0.0418 (6)	
H13A	0.7678	1.1756	−0.0216	0.050*	
H13B	0.6174	1.1634	−0.0745	0.050*	
C14	0.0690 (4)	1.3023 (3)	0.0107 (3)	0.0748 (10)	
H14A	0.0055	1.2405	0.0349	0.112*	
H14B	0.0458	1.3466	−0.0617	0.112*	
H14C	0.0409	1.3694	0.0489	0.112*	
C15	0.6104 (3)	1.3283 (3)	0.1027 (2)	0.0520 (7)	
H15A	0.7093	1.2782	0.1434	0.078*	
H15B	0.5504	1.4037	0.1290	0.078*	
H15C	0.6378	1.3616	0.0318	0.078*	
C16	0.4492 (3)	1.1912 (3)	0.2195 (2)	0.0495 (7)	
H16A	0.5431	1.1463	0.2654	0.074*	
H16B	0.3871	1.1296	0.2231	0.074*	
H16C	0.3818	1.2697	0.2396	0.074*	

### Atomic displacement parameters (Å^2^)

**Table:**

	*U*^11^	*U*^22^	*U*^33^	*U*^12^	*U*^13^	*U*^23^
Cl1	0.0542 (5)	0.0882 (6)	0.0592 (5)	−0.0075 (4)	−0.0185 (4)	−0.0037 (4)
Cl2	0.0491 (4)	0.0642 (5)	0.0495 (4)	−0.0115 (4)	−0.0061 (3)	−0.0160 (3)
O1W	0.0910 (19)	0.115 (2)	0.105 (2)	−0.0405 (17)	−0.0193 (16)	−0.0280 (17)
O2W	0.0793 (17)	0.102 (2)	0.0905 (18)	−0.0236 (15)	−0.0020 (14)	−0.0391 (15)
O3W	0.0810 (17)	0.0622 (15)	0.112 (2)	−0.0201 (13)	0.0023 (15)	−0.0176 (14)
N1	0.0298 (11)	0.0359 (13)	0.0439 (12)	−0.0045 (9)	−0.0044 (9)	−0.0161 (10)
N2	0.0287 (11)	0.0356 (12)	0.0376 (12)	−0.0097 (10)	−0.0039 (9)	−0.0109 (10)
N3	0.0308 (11)	0.0423 (13)	0.0488 (13)	−0.0118 (10)	−0.0020 (9)	−0.0169 (10)
N4	0.0335 (11)	0.0338 (12)	0.0365 (12)	−0.0138 (10)	−0.0049 (10)	−0.0096 (9)
C1	0.0317 (13)	0.0498 (16)	0.0449 (15)	−0.0112 (12)	0.0008 (11)	−0.0201 (13)
C2	0.0345 (14)	0.0353 (15)	0.0347 (14)	−0.0032 (11)	0.0008 (11)	−0.0101 (11)
C3	0.0444 (15)	0.0318 (14)	0.0508 (16)	−0.0092 (12)	0.0013 (12)	−0.0152 (12)
C4	0.0386 (14)	0.0360 (14)	0.0412 (14)	−0.0130 (11)	−0.0019 (11)	−0.0177 (11)
C5	0.0383 (14)	0.0452 (15)	0.0387 (14)	−0.0180 (12)	0.0040 (11)	−0.0137 (12)
C6	0.0405 (16)	0.0421 (17)	0.100 (3)	0.0022 (14)	−0.0053 (16)	−0.0044 (17)
C7	0.0495 (16)	0.0565 (17)	0.0431 (16)	−0.0176 (14)	0.0012 (13)	−0.0214 (13)
C8	0.0513 (17)	0.0551 (18)	0.0588 (18)	−0.0248 (14)	0.0019 (14)	−0.0283 (15)
C9	0.0345 (14)	0.0575 (17)	0.0491 (16)	−0.0210 (13)	0.0061 (12)	−0.0249 (13)
C10	0.0345 (14)	0.0445 (16)	0.0371 (14)	−0.0064 (12)	−0.0045 (11)	−0.0139 (12)
C11	0.0440 (15)	0.0339 (14)	0.0525 (17)	−0.0050 (12)	−0.0091 (12)	−0.0117 (12)
C12	0.0394 (14)	0.0337 (14)	0.0460 (15)	−0.0102 (11)	−0.0045 (12)	−0.0164 (12)
C13	0.0436 (15)	0.0476 (16)	0.0419 (15)	−0.0244 (13)	0.0028 (12)	−0.0127 (12)
C14	0.0463 (18)	0.063 (2)	0.114 (3)	0.0041 (16)	−0.0190 (18)	−0.041 (2)
C15	0.0553 (17)	0.0420 (16)	0.0684 (19)	−0.0194 (14)	−0.0042 (15)	−0.0239 (14)
C16	0.0498 (16)	0.0545 (17)	0.0488 (17)	−0.0144 (14)	−0.0010 (13)	−0.0226 (14)

### Geometric parameters (Å, º)

**Table:**

O1W—H1W1	0.8197	C6—H6A	0.9600
O1W—H2W1	0.8227	C6—H6B	0.9600
O2W—H1W2	0.8219	C6—H6C	0.9600
O2W—H2W2	0.8240	C7—H7A	0.9600
O3W—H1W3	0.8259	C7—H7B	0.9600
O3W—H2W3	0.8234	C7—H7C	0.9600
N1—C2	1.265 (3)	C8—H8A	0.9600
N1—C1	1.457 (3)	C8—H8B	0.9600
N2—C5	1.486 (3)	C8—H8C	0.9600
N2—C4	1.525 (3)	C9—C13^ii^	1.504 (4)
N2—H1N2	0.92 (3)	C9—H9A	0.9700
N2—H2N2	0.90 (2)	C9—H9B	0.9700
N3—C10	1.260 (3)	C10—C14	1.495 (4)
N3—C9	1.459 (3)	C10—C11	1.509 (3)
N4—C13	1.489 (3)	C11—C12	1.532 (3)
N4—C12	1.518 (3)	C11—H11A	0.9700
N4—H1N4	0.93 (3)	C11—H11B	0.9700
N4—H2N4	0.86 (2)	C12—C15	1.517 (3)
C1—C5^i^	1.508 (3)	C12—C16	1.522 (4)
C1—H1A	0.9700	C13—C9^ii^	1.504 (4)
C1—H1B	0.9700	C13—H13A	0.9700
C2—C6	1.494 (3)	C13—H13B	0.9700
C2—C3	1.507 (3)	C14—H14A	0.9600
C3—C4	1.523 (3)	C14—H14B	0.9600
C3—H3A	0.9700	C14—H14C	0.9600
C3—H3B	0.9700	C15—H15A	0.9600
C4—C8	1.522 (3)	C15—H15B	0.9600
C4—C7	1.523 (4)	C15—H15C	0.9600
C5—C1^i^	1.508 (3)	C16—H16A	0.9600
C5—H5A	0.9700	C16—H16B	0.9600
C5—H5B	0.9700	C16—H16C	0.9600
			
H1W1—O1W—H2W1	98.0	H7A—C7—H7C	109.5
H1W2—O2W—H2W2	101.4	H7B—C7—H7C	109.5
H1W3—O3W—H2W3	105.5	C4—C8—H8A	109.5
C2—N1—C1	120.7 (2)	C4—C8—H8B	109.5
C5—N2—C4	118.08 (19)	H8A—C8—H8B	109.5
C5—N2—H1N2	106.4 (16)	C4—C8—H8C	109.5
C4—N2—H1N2	109.7 (16)	H8A—C8—H8C	109.5
C5—N2—H2N2	106.5 (15)	H8B—C8—H8C	109.5
C4—N2—H2N2	105.8 (15)	N3—C9—C13^ii^	110.7 (2)
H1N2—N2—H2N2	110 (2)	N3—C9—H9A	109.5
C10—N3—C9	120.1 (2)	C13^ii^—C9—H9A	109.5
C13—N4—C12	118.30 (19)	N3—C9—H9B	109.5
C13—N4—H1N4	108.3 (17)	C13^ii^—C9—H9B	109.5
C12—N4—H1N4	106.2 (16)	H9A—C9—H9B	108.1
C13—N4—H2N4	106.8 (15)	N3—C10—C14	124.9 (2)
C12—N4—H2N4	104.3 (15)	N3—C10—C11	119.1 (2)
H1N4—N4—H2N4	113 (2)	C14—C10—C11	116.0 (2)
N1—C1—C5^i^	110.30 (19)	C10—C11—C12	116.8 (2)
N1—C1—H1A	109.6	C10—C11—H11A	108.1
C5^i^—C1—H1A	109.6	C12—C11—H11A	108.1
N1—C1—H1B	109.6	C10—C11—H11B	108.1
C5^i^—C1—H1B	109.6	C12—C11—H11B	108.1
H1A—C1—H1B	108.1	H11A—C11—H11B	107.3
N1—C2—C6	126.2 (2)	C15—C12—N4	109.0 (2)
N1—C2—C3	119.0 (2)	C15—C12—C16	110.2 (2)
C6—C2—C3	114.9 (2)	N4—C12—C16	106.2 (2)
C2—C3—C4	118.2 (2)	C15—C12—C11	110.4 (2)
C2—C3—H3A	107.8	N4—C12—C11	109.54 (19)
C4—C3—H3A	107.8	C16—C12—C11	111.3 (2)
C2—C3—H3B	107.8	N4—C13—C9^ii^	109.9 (2)
C4—C3—H3B	107.8	N4—C13—H13A	109.7
H3A—C3—H3B	107.1	C9^ii^—C13—H13A	109.7
C8—C4—C3	110.6 (2)	N4—C13—H13B	109.7
C8—C4—C7	110.3 (2)	C9^ii^—C13—H13B	109.7
C3—C4—C7	111.4 (2)	H13A—C13—H13B	108.2
C8—C4—N2	108.9 (2)	C10—C14—H14A	109.5
C3—C4—N2	109.49 (19)	C10—C14—H14B	109.5
C7—C4—N2	106.1 (2)	H14A—C14—H14B	109.5
N2—C5—C1^i^	109.9 (2)	C10—C14—H14C	109.5
N2—C5—H5A	109.7	H14A—C14—H14C	109.5
C1^i^—C5—H5A	109.7	H14B—C14—H14C	109.5
N2—C5—H5B	109.7	C12—C15—H15A	109.5
C1^i^—C5—H5B	109.7	C12—C15—H15B	109.5
H5A—C5—H5B	108.2	H15A—C15—H15B	109.5
C2—C6—H6A	109.5	C12—C15—H15C	109.5
C2—C6—H6B	109.5	H15A—C15—H15C	109.5
H6A—C6—H6B	109.5	H15B—C15—H15C	109.5
C2—C6—H6C	109.5	C12—C16—H16A	109.5
H6A—C6—H6C	109.5	C12—C16—H16B	109.5
H6B—C6—H6C	109.5	H16A—C16—H16B	109.5
C4—C7—H7A	109.5	C12—C16—H16C	109.5
C4—C7—H7B	109.5	H16A—C16—H16C	109.5
H7A—C7—H7B	109.5	H16B—C16—H16C	109.5
C4—C7—H7C	109.5		
			
C2—N1—C1—C5^i^	−157.0 (2)	C10—N3—C9—C13^ii^	169.2 (2)
C1—N1—C2—C6	2.0 (4)	C9—N3—C10—C14	0.6 (4)
C1—N1—C2—C3	−178.3 (2)	C9—N3—C10—C11	−179.0 (2)
N1—C2—C3—C4	22.6 (3)	N3—C10—C11—C12	−37.1 (4)
C6—C2—C3—C4	−157.6 (2)	C14—C10—C11—C12	143.3 (3)
C2—C3—C4—C8	−175.2 (2)	C13—N4—C12—C15	−55.2 (3)
C2—C3—C4—C7	61.8 (3)	C13—N4—C12—C16	−174.0 (2)
C2—C3—C4—N2	−55.2 (3)	C13—N4—C12—C11	65.7 (3)
C5—N2—C4—C8	58.2 (3)	C10—C11—C12—C15	177.6 (2)
C5—N2—C4—C3	−62.8 (3)	C10—C11—C12—N4	57.5 (3)
C5—N2—C4—C7	176.9 (2)	C10—C11—C12—C16	−59.7 (3)
C4—N2—C5—C1^i^	−177.59 (19)	C12—N4—C13—C9^ii^	178.0 (2)

Symmetry codes: (i) −*x*+2, −*y*+1, −*z*+1; (ii) −*x*+1, −*y*+2, −*z*.

### Hydrogen-bond geometry (Å, º)

**Table:**

*D*—H···*A*	*D*—H	H···*A*	*D*···*A*	*D*—H···*A*
N2—H2*N*2···N1	0.90 (3)	2.02 (3)	2.732 (3)	135.6 (18)
N4—H2*N*4···N3	0.86 (3)	2.03 (3)	2.740 (3)	140 (2)
O1*W*—H2*W*1···Cl1	0.82	2.54	3.343 (4)	167
O2*W*—H2*W*2···Cl1	0.82	2.51	3.324 (3)	168
O3*W*—H1*W*3···Cl1	0.83	2.53	3.298 (3)	156
O3*W*—H2*W*3···Cl2	0.82	2.32	3.138 (3)	175
N2—H1*N*2···Cl1	0.92 (3)	2.28 (3)	3.201 (3)	177 (3)
N4—H1*N*4···Cl2	0.93 (3)	2.27 (3)	3.178 (3)	167 (2)
O1*W*—H1*W*1···Cl2^iii^	0.82	2.49	3.282 (3)	163
O2*W*—H1*W*2···O3*W*^iii^	0.82	2.16	2.955 (4)	161
C9—H9*B*···Cl2^iii^	0.97	2.75	3.709 (3)	172

Symmetry code: (iii) *x*−1, *y*, *z*.
